# Novel Filoviruses, Hantavirus, and Rhabdovirus in Freshwater Fish, Switzerland, 2017

**DOI:** 10.3201/eid2712.210491

**Published:** 2021-12

**Authors:** Melanie M. Hierweger, Michel C. Koch, Melanie Rupp, Piet Maes, Nicholas Di Paola, Rémy Bruggmann, Jens H. Kuhn, Heike Schmidt-Posthaus, Torsten Seuberlich

**Affiliations:** University of Bern, Bern, Switzerland (M.M. Hierweger, M.C. Koch, M. Rupp, R. Bruggmann, H. Schmidt-Posthaus, T. Seuberlich); KU Leuven, Leuven, Belgium (P. Maes);; US Army Medical Research Institute of Infectious Diseases, Frederick, Maryland, USA (N. Di Paola);; Integrated Research Facility at Fort Detrick, Frederick (J. H. Kuhn)

**Keywords:** European perch, filovirus, hantavirus, rhabdovirus, freshwater fish, viruses, Switzerland

## Abstract

European perch (*Perca fluviatilis*) are increasingly farmed as a human food source. Viral infections of European perch remain largely unexplored, thereby putting farm populations at incalculable risk for devastating fish epizootics and presenting a potential hazard to consumers. To address these concerns, we applied metatranscriptomics to identify disease-associated viruses in European perch farmed in Switzerland. Unexpectedly, in clinically diseased fish we detected novel freshwater fish filoviruses, a novel freshwater fish hantavirus, and a previously unknown rhabdovirus. Hantavirus titers were high, and we demonstrated virus in macrophages and gill endothelial cells by using in situ hybridization. Rhabdovirus titers in organ samples were low, but virus could be isolated on cell culture. Our data add to the hypothesis that filoviruses, hantaviruses, and rhabdoviruses are globally distributed common fish commensals, pathogens, or both. Our findings shed new light on negative-sense RNA virus diversity and evolution.

Aquaculture is the fastest growing sector in food production worldwide (*1*); innovative intensive production technologies, such as recirculating aquaculture systems (RAS), are becoming increasingly valuable for commercial fish production (*2*). Attempts are continually made to introduce new types of fish into aquaculture, to reduce overfishing of wild fish populations, and to satisfy the progressing consumer demand for diverse supply (*3*). One example is the adaptation of European perch (*Perca fluviatilis*; order Perciformes, family Percidae) to farming and the growing consumer interest in this fish (*4*). European perch are actinopterygiids that naturally inhabit slow-flowing rivers, lakes, or ponds in Europe and northern Asia. During the 19th century, they were introduced into Australia as angling fish, where they are now considered invasive, competing with native fish for food and space, preying on other fish, and breeding to overpopulation (*5*).

Although little is known about diseases affecting European perch in the wild, infectious diseases lead to high mortality among these fish in aquaculture, making farming of these fish economically challenging. One of the main threats is infection with perch rhabdovirus (PRV; family *Rhabdoviridae*, genus *Perhabdovirus*), leading to the central nervous system (CNS) signs of loss of equilibrium and aberrant swimming behavior and to higher mortality (*2*,*6*–*10*). Lack of investigation of the occurrence and diversity of other pathogenic virus infections of European perch that result in disease impairs the treatment, control, and prevention of disease outbreaks in farm populations. To address this knowledge gap, we applied virus diagnostics, including metatranscriptomics (virus discovery by high-throughput RNA sequencing and bioinformatics), to a set of samples collected from sick juvenile European perch at a perch farm in Switzerland in 2017. Although we did not find PRV in these fish, our investigation led to the discovery of 5 novel negative-sense RNA viruses, belonging to the negarnaviricot families *Rhabdoviridae*, *Filoviridae*, and *Hantaviridae*, that could possibly contribute to disease development.

## Methods

### European Perch Origin

The European perch used in this study were raised in a private pond in Saxony, Germany, and were exported to a farm that uses RAS in Bernese Oberland, Switzerland, at 16 g (≈11,200 fish) and 33 g (≈4,800 fish). Eleven live juvenile European perch were sent to the Centre for Fish and Wildlife Health (FIWI), University of Bern (Bern, Switzerland), where they were euthanized and subjected to microbiological (including parasitologic, bacteriologic, mycologic, and virologic) and pathologic (including histopathologic) examination. Because the remaining fish at the farm exhibited clinical signs of disease, they were kept in quarantine for an additional 2 months and subsequently euthanized.

### Cell Culture

We used bluegill (*Lepomis macrochirus*) fry cells (BF-2) and fathead minnow (*Pimephales promelas*) epithelioma papulosum cyprini (EPC) cells. These cells were originally obtained from the Friedrich-Loeffler-Institute, Federal Research Institute for Animal Health (Greifswald-Insel Riems, Germany; Collection of Cell Lines in Veterinary Medicine, catalog nos. CCLV-RIE 290 and CCLV-RIE 173 are maintained at FIWI).

We inoculated the cells with small pieces of pooled CNS or spleen, kidney, heart, and pyloric ceca tissue from 5 of the 11 euthanized European perch (in total <5 g) and incubated at 15°C. We monitored the cell cultures for CPE daily for 7 days by using light microscopy and then monitored subcultures for another 7 days. We harvested supernatants from cell cultures showing CPE and tested by reverse transcription PCR (RT-PCR) for the common European perch pathogen PRV, targeting the glycoprotein (*G*) gene of PRV isolate 9574.1 (GenBank accession no. JF502613), according to a previously published protocol, by using the primer pair oPVP116/118 and oPVP126/Rha2 (*11*).

### High-Throughput Sequencing and Bioinformatics

We extracted total RNA from fresh-frozen pooled visceral organs and CNS tissue, originally taken for cell culture inoculation by using TRI Reagent (Sigma Life Sciences, https://www.sigmaaldrich.com), according to the manufacturer’s instructions. We then prepared a high-throughput sequencing (HTS) library with the TruSeq Stranded Total RNA kit (Illumina, https://www.illumina.com) and performed HTS on a HiSeq 3000 machine (Illumina), generating paired-end reads of 2 × 150 bp. We performed bioinformatic analysis as described previously (*12*) (Appendix).

### Reverse Transcription PCR, Rapid Amplification of cDNA Ends, and Sanger Sequencing

To fill gaps between HTS scaffolds, we reverse-transcribed extracted RNA to cDNA, performed PCRs, and subjected the amplicons to Sanger sequencing (Appendix). We performed 3′ and 5′ rapid amplification of cDNA ends (RACE), as described previously, on RNA extracted from pooled organs and CNS tissue as well as cell culture supernatants (*13*) (Appendix). 

### Taxonomic Analyses

We performed taxonomic analyses by using protein or nucleic acid sequences, following precedents established by the International Committee on Taxonomy of Viruses (ICTV) *Filoviridae* (*14*,*15*), *Hantaviridae* (*16*), and *Rhabdoviridae* (*17*) Study Groups. New filovirus-like genome sequences were analyzed by using PAirwise Sequence Comparison (PASC) (*18*) and maximum-likelihood phylogenetics. Filovirus phylogenetic estimations were inferred in FastTree version 2.1 (*19*) by using a general time-reversible model with 20 gamma-rate categories, 5,000 bootstrap replicates, and exhaustive search parameters (-slow) and pseudocounts (-pseudo). The new rhabdovirus-like sequence was taxonomically placed via analysis of its full-length large protein gene (*L*) sequence using maximum-likelihood phylogenetics. We applied the maximum-likelihood method in MEGA X (*20*) with 1,000 bootstraps for rhabdovirus and hantavirus phylogenetic estimations.

### Histopathology and In Situ Hybridization

During a complete necropsy of the 11 fish, we used 3 whole perch for histologic examination. We fixed these fish in 10% buffered formalin for 24 hours and embedded cut sections of the gills, longitudinal head sections, and longitudinal cut sections of the body cavity in paraffin. We prepared 3-μm sections and stained them with hematoxylin and eosin according to standard protocols. We conducted chromogenic in situ hybridization on all formalin-fixed paraffin-embedded (FFPE) tissues used for histopathology. We performed staining with the RNAscope system (Advanced Cell Diagnostics, https://acdbio.com) (Appendix).

## Results

### Clinical and Pathologic Findings

In December 2016, a total of 16,000 juvenile European perch were imported from Saxony, Germany, to a farm using RAS in Bernese Oberland, Switzerland. After arrival, the fish were routinely quarantined. Shortly after arrival, because of a high death rate of 1% per day (reference range 0.01%–0.03% per day), 11 randomly selected live fish were sent to FIWI for microbiological and pathologic examination. Clinical signs included anorexia, lethargy, skin ulcerations, multifocal hemorrhages, and eroded tail fins. Culture and PCR indicated that skin ulcerations were caused by oomycete (*Saprolegnia parasitica*) infection (*21*). Histopathology revealed mild to moderate gill epithelial proliferation and epithelial cell hypertrophy. Additional findings included necrotizing dermatitis with hemorrhage and intralesional oomycete hyphi and bacterial colonies. The newly arrived fish were treated with flubendazole, formalin, and peracetic acid, but deaths increased to 1.8%–4.2% per day. Two months after importation, deaths for the quarantined fish reached 22%–27% in total. All remaining fish were euthanized and discarded, thereby preventing these fish from entering the food chain.

### Virus Isolation

For routine virus investigation, we exposed standard fish cell cultures (BF-2 and EPC) to suspensions of pooled perch CNS and pooled visceral organs. We selected these cell lines because of their high susceptibility to diverse fish viruses (*22*). CPE developed 10 days after inoculation of CNS suspension into BF-2 cells and 13 days after inoculation into EPC cells. Affected cell supernatants were harvested and tested preliminarily positive for PRV infection by RT-PCR. The sequence of the detected amplicon was, however, only 78% identical to the sequence of the perhabdovirus lake trout rhabdovirus (LTRV; GenBank accession no. AF434991), indicating the presence of a perhabdovirus distinct from PRV/ LTRV.

### Novel Perhabdovirus

**Figure 1:**
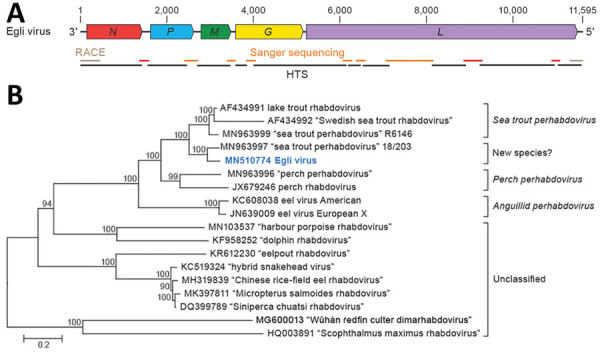
Identifying a novel rhabdovirus in European perch. A) Schematic representation of the EGLV genome organization; open reading frames are indicated by colored arrows. B) Maximum-likelihood phylogenetic tree of the nucleotide sequence of the EGLV *L* gene (bold blue) and representative classified and unclassified members of the genus *Perhabdovirus.* Numbers near nodes on the trees indicate bootstrap values. Branches are labeled by GenBank accession number and virus name. Names of unclassified likely perhabdoviruses are placed in quotation marks. Scale bar indicates number of substitutions per site, reflected by branch lengths. EGLV, Egli virus; *G*, glycoprotein gene; HTS, high-throughput sequencing; *L*, large protein gene; *M*, matrix protein gene; *N*, nucleoprotein gene; *P*, phosphoprotein gene; RACE, rapid amplification of cDNA ends.

To further characterize the putative novel perhabdovirus, we performed metatranscriptomics by using HTS and bioinformatic analysis of pooled CNS tissue and visceral organ RNA extracts of 5 fish. We found 16 sequence scaffolds 247–1,454-nt long with mean k-mer coverages of 1.0–5.9 and nucleotide sequence identities of 62%–98% to perhabdovirus genomes (Appendix Table 1). We mapped these scaffolds to LTRV (GenBank accession no. AF434991) and PRV (GenBank accession no. JX679246) as references and closed sequence gaps by RT-PCR and RACE followed by Sanger sequencing. The resulting complete genome of the novel virus was 11,595-nt long and had the characteristic genomic organization of (pe)rhabdoviruses (Figure 1, panel A). Each of the open reading frames (ORFs) is flanked by conserved transcriptional initiation (3′-UUGUUC) and termination/polyadenylation (3′-AURC[U]_7_) signals with inverse complementarity of 13 nt of the 3′ and 5′ terminal genome sequences.

Phylogenetic comparison of the *L* gene confirmed a close relationship of the new virus to the 2 members of the species *Sea trout perhabdovirus* (i.e., LTRV and Swedish sea trout virus; GenBank accession no. AF434992) (Figure 1, panel B) and a recently described and thus far unclassified virus from a percid (sea trout rhabdovirus isolate 18/203; GenBank accession no. MN963997). This relationship is also reflected in phylogenetic comparisons of the nucleoprotein (*N*), phosphoprotein (*P*), matrix protein (*M*), and *G* genes (Appendix Figure 1). The current perhabdovirus sequence-based species demarcation criterion is a minimum divergence of 15% in the *L* gene (*17*). The *L* gene sequence of the novel virus is most closely related to that of the isolate 18/203 (divergence 10%); *L* genes of both viruses are ≈19% divergent from the next closest related perhabdovirus, sea trout perhabdovirus isolate R6146 (GenBank accession no. MN963999), thus indicating that we discovered a novel perhabdovirus that should be assigned to a new species along with isolate 18/203. We named the new virus Egli (EGLV) virus after a local Swiss-German word for European perch and deposited the complete viral genome sequence into GenBank (accession no. MN510774). Detection of EGLV in European perch FFPE tissue sections by RNA in situ hybridization was unsuccessful.

### Four Novel Filoviruses

In addition to scaffolds that ultimately led us to identify EGLV, we found 41 scaffolds 240–4,726-nt long with low k-mer coverage (0.8–4.8×). The deduced amino acid sequences were 28%–30% identical to proteins of Huángjiāo virus (HUJV), a recently identified virus of marine greenfin horse‐faced filefish (*Thamnaconus septentrionalis*) captured in the East China Sea (*23*) (Appendix Table 1). Alignment of these scaffolds to the HUJV genome (GenBank accession no. MG599981) and the deduced amino acid sequences to those of HUJV-encoded proteins revealed a complex scenario suggesting the presence of several distinct thamnoviral genomes, however with numerous gaps between the scaffolds. To obtain complete or coding-complete viral genome sequences, we resequenced the HTS library to generate ≈10 times more paired-end reads (2,051,046,671) than during the initial HTS run, reassembled the sequences, and performed RT-PCR and Sanger sequencing to bridge sequence gaps. This effort resulted in 3 long scaffolds of 13,764 nt (k-mer coverage 24×), 14,593 nt (k-mer coverage 15×), and 13,066 nt (k-mer coverage 5×), corresponding to 3 novel viruses.

**Figure 2:**
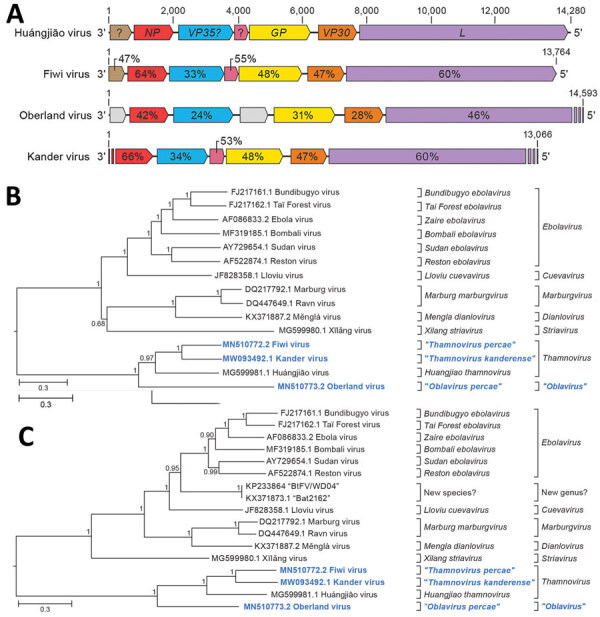
Identifying 3 novel filoviruses in European perch. A) Schematic representation of the genome organization of Fiwi virus, Oberland virus, and Kander virus compared with Huángjiāo virus (HUJV). Open reading frames (ORFs) are indicated by colored arrows. ORFs encoding HUJV-like proteins are depicted by the same color and sequence similarities are indicated as percentages. Undetermined ORF starts and ends are shown as stripes. B, C) Maximum-likelihood phylogenetic trees of the new filovirus genome sequences (bold blue) generated by using coding-complete and near-complete genome sequences (B) or only *L* gene sequences (C) of representative members of the family *Filoviridae*. Numbers near nodes on the trees indicate bootstrap values. Branches are labeled by GenBank accession number, and virus name. Scale bar indicates number of substitutions per site, reflected by branch lengths. *GP*, glycoprotein gene; *L*, large protein gene; *NP*, nucleoprotein gene; *VP30*, transcriptional activator gene; *VP35*, polymerase cofactor gene.

We named these viruses Fiwi virus (FIWIV; GenBank accession no. MN510772), after FIWI; Oberland virus (OBLV; GenBank accession no. MN510773), after Bernese Oberland; and Kander virus (KNDV; GenBank accession no. MW093492), after the Kander River, which flows through Bernese Oberland. Whereas the FIWIV genome appears to be coding complete, the sequences of OBLV are coding incomplete at the 5′ terminus and of KNDV at both the 5′ and the 3′ termini. All attempts to determine the authentic 3′ and 5′ termini by RACE were unsuccessful, most likely because of low viral RNA loads. However, all 3 sequences have the genomic features of HUJV, encoding the filovirus-typical proteins nucleoprotein (NP), polymerase cofactor (VP35), glycoprotein (GP_1,2_), transcriptional activator (VP30), and large protein (L) containing an RNA-directed RNA polymerase (RdRp) domain, as well as 1–2 novel proteins (*14*,*23*,*24*) (Figure 2, panel A). Phylogenetic comparison of the FIWIV, OBLV, and KNDV genomic sequences (Figure 2, panel B) and *L* gene sequences with those of representative classified viruses of the family *Filoviridae* (Figure 2, panel C) confirmed the genetic relationship of all 3 viruses to HUJV. The current demarcation criteria for filovirus sequence-based genus and species are >55% and >23% sequence divergence over complete genome sequences determined by using PASC (*14*,*15*). We found a pairwise divergence of 49% compared with HUJV by using the FIWIV genome sequence (Table), indicating that FIWIV is a member of a new thamnovirus species (“*Thamnovirus percae*”). The available KNDV genome sequence is 49% divergent from HUJV and 37% divergent from FIWIV (Table), suggesting that KNDV represents yet another novel thamnovirus species (“*Thamnovirus kanderense*”). In contrast, OBLV was >62% divergent from HUJV, FIWIV, and KNDV viruses and thus represents a new species (“*Oblavirus percae*”) within a new genus (“*Oblavirus*”). All attempts to detect FIWIV or OBLV viruses in 3 European perch FFPE tissue sections were unsuccessful.

**Table:** Pairwise distances of complete or coding-complete genome nucleotide sequences between the newly identified Fiwi virus, Huángjiāo virus, and the closest related mammalian filovirus, Bombali virus*

Virus	Virus			
	Fiwi	Oberland	Kander	Huángjiāo
Oberland	66%			
Kander	37%	62%		
Huángjiāo	49%	64%	49%	
Bombali	86%	87%	87%	86%

*Fiwi virus, GenBank accession no. MN510772; Oberland virus, GenBank accession no. MN510773; Kander virus, GenBank accession no. MW093492; Bombali virus, GenBank accession no.MK340750. Oberland and Kander virus sequences are not coding complete and were compared on the basis of the available incomplete sequences.

In addition to FIWIV, OBLV, and KNDV, we found 4 shorter scaffolds (573–3,259 nt; Appendix Figure 2) similar to various HUJV genes, but attempts to demonstrate a physical linkage of these sequences by RT-PCR were not successful. Still, the presence of these scaffolds is indicative of at least 1 additional novel filovirus, which we were not able to further characterize.

### Novel Hantavirus

**Figure 3:**
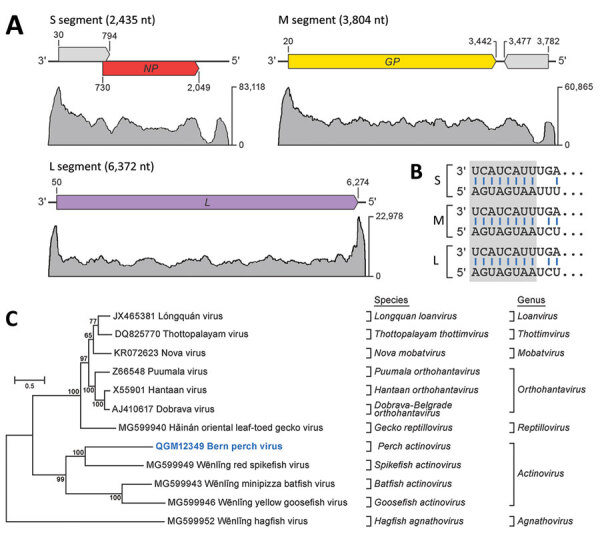
Identifying a novel hantavirus in European perch. A) Schematic representation of the 3 genome segments of Bern perch virus; open reading frames are indicated as colored arrows. Coverage plots of high-throughput sequencing reads are shown for each segment, and maximum-read coverages are indicated on the right. B) Alignment of the terminal sequences (11 nt) of the 3 segments. The terminal 8 nucleotides (gray box) are complementary within and conserved among segments. C) Maximum-likelihood phylogenetic tree of the Bern perch virus RNA-directed RNA-polymerase amino-acid sequence (bold blue) with RNA-directed RNA-polymerase amino-acid sequences of representative members of the family *Hantaviridae.* Numbers near nodes on the trees indicate bootstrap values. Branches are labeled by GenBank accession number, and virus name. Scale bar indicates number of substitutions per site, reflected by branch lengths. *GP*, glycoprotein gene; L, large; M, medium; S, small; *NP*, nucleocapsid protein gene.

In the same RNA extract of pooled organs, we found 3 scaffolds, of which the deduced amino acid sequences were 25%–35% similar to those of the large (L), medium (M), and small (S) segments of Wēnlǐng minipizza batfish virus (WEMBV) and Wēnlǐng red spikefish virus (WERSV). WEMBV was recently identified in apparently healthy minipizza batfish (*Halieutaea stellate*) and WERSV in red spikefish (*Triacanthodes anomalus*) captured in the East China Sea (Appendix Table 1) (*23*). The mean k-mer coverage of these scaffolds ranged from 3 × 10^3^ to 1.5 × 10^4^, indicating a high viral RNA load. Using Sanger sequencing and RACE, we determined the complete sequences of the genomic L (GenBank accession no. MN510769), M (GenBank accession no. MN510770), and S (GenBank accession no. MN510771) segments of a novel virus, here named Bern perch virus. Similar to WEMBV and WERSV, the Bern perch virus (BPV) L segment (6,372 nt) was deduced to encode the L protein including an RdRp domain, the M segment (3,804 nt) was deduced to encode the glycoprotein precursor, and the S segment (2,435 nt) was deduced to encode the nucleocapsid protein (Figure 3, panel A). The S and M segments contain 2 additional ORFs encoding putative proteins not represented in current protein databases in antisense (S segment) and sense (M segment) orientation. Alignment of the 3′ and 5′ sequences of all 3 segments revealed that the 8 terminal nucleotides are complementary within and conserved among segments (Figure 3, panel B), a known feature of members of the order *Bunyavirales* (*25*). However, these terminal sequences differ from those of members of the genus *Orthohantavirus* and are similar to those of members of the genus *Orthobunyavirus* (Appendix Table 1). Phylogenetic analysis of the L protein confirmed the close relationship of Bern perch virus to all currently classified actinoviruses (Figure 3, panel C; Appendix Figure 3) but indicated the need for a novel species to accommodate this virus. This need was confirmed by DEmARC (Diversity Partitioning by Hierarchical Clustering) analysis (*26*); on the basis of this evidence, the ICTV officially established this species as *Perch actinovirus* in 2021 (*27*,*28*).

**Figure 4:**
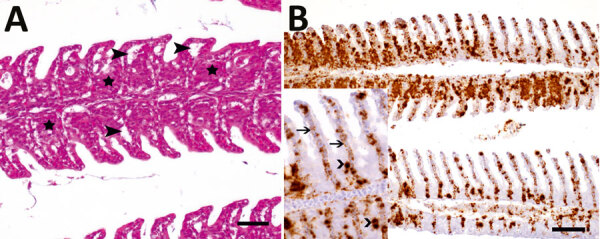
Histopathologic lesions and viral RNA in European perch infected with Bern perch virus. A) Histopathologic lesions in gills (hematoxylin and eosin stain) showing epithelial hypertrophy and hyperplasia, multifocally leading to lamellar fusion (stars) and multifocal epithelial lifting due to edema (closed arrowheads). Scale bar indicates 25 μm. B) In situ hybridization detection of Bern perch virus RNA in gills (brown labeling): positive macrophages, more pronounced in proliferated areas, and endothelial cells. Inset: higher magnification showing positive macrophages (open arrowheads) and endothelial cells (arrows with open heads). Scale bar indicates 50 μm.

Using in situ hybridization on FFPE tissue sections, we were able to detect Bern perch virus genomic RNA in gills with histopathologic lesions of 2 fish (Figure 4, panels A, B; Appendix Figure 4) and in a granuloma in the perivisceral fat tissue of 1 of these animals. Morphologically, we identified the affected cells in the gills and the perivisceral fat tissue as putative macrophages. In addition, putative endothelial cells were labeled positively in the gills (Figure 4, panel B).

## Discussion

The diversity of fish viruses, in particular that of RNA viruses, remains poorly understood (*29*). Recent initial studies indicate that this diversity is enormous and that many viral taxa that have been established for pathogens of humans and other mammals need to be redefined (*23*,*30*–*32*). Husbandry conditions in huge tanks used on farms may favor the emergence and rapid intraspecies and interspecies transmission of fish viruses, potentially resulting in high economic loss for the fish industry. Also of concern is the introduction of novel viruses from fish farms into native fauna, which could have disastrous ecologic consequences. In addition, viruses of unknown pathogenicity in food animals may have zoonotic potential of yet unpredictable importance.

In this study, we identified 1 novel rhabdovirus, 4 novel filoviruses (3 confirmed and 1 likely), and 1 novel hantavirus in morbid farmed European perch. The discovery of a novel rhabdovirus was not surprising and adds to the role of rhabdoviruses in fish health; rhabdoviruses, in particular those of the genera *Novirhabdovirus*, *Perhabdovirus*, *Sprivivirus*, and *Vesiculovirus*, are notorious marine and freshwater fish pathogens, causing diseases characterized by high lethality (*33*,*34*). Thus, these viruses pose a considerable threat to aquaculture. The European perch examined in this study exhibited signs compatible with rhabdovirus infection (*7*). Using HTS and exposing cell cultures to CNS tissue suspensions, we discovered a novel perhabdovirus, Egli virus. Although the viral RNA loads in tissues were low and we could not detect RNA by in situ hybridization in tissue sections, we were able to isolate the virus from brain tissue. Rhabdoviruses in perch are usually associated with disease and not known as commensals. The host range of Egli virus is unknown, but genetically it is more closely related to viruses identified in trout than to those infecting perch, suggesting the possibility of cross-species transmission, highlighting a concern for farms that raise fish other than perch and for native fish populations. Discovery of a lake trout rhabdovirus as a probable cause of disease in European perch in Ireland (*7*) supports the hypothesis of high potential for interspecies transmission of these viruses.

Unexpectedly, we were able to assemble near-complete viral genomes of 3 novel filoviruses (Fiwi, Oberland, and Kander viruses) and detected a likely fourth filovirus in the diseased perch. Until recently, filoviruses, notorious for causing disease in humans with extremely high lethality (*35*), were thought to exclusively infect mammals. This view changed with the discovery of Xīlǎng virus (the only member of genus *Striavirus*) in striated frogfish (*Antennarius striatus* ) and HUJV in greenfin horse‐faced filefish captured in the East China Sea (*23*) as well as apparent thamnoviruses John Dory filovirus in John Dory (*Zeus faber*) and blue spotted goatfish filovirus in blue spotted goatfish (*Upeneichthys lineatus*) purchased at a fish market in Sydney, New South Wales, Australia (*31*). However, currently, <300 nt contigs are known from John Dory fish and blue spotted goatfish filoviruses; hence, their true taxonomic affiliation remains to be determined. In contrast to Xīlǎng virus, HUJV, John Dory filovirus, and blue spotted goatfish filovirus (all of which were found in marine fish in China and Australia), FIWIV, OBLV, and KNDV apparently infect freshwater fish in Europe. This geographic and ecologic distribution indicates that filoviruses are broadly dispersed fish commensals or potential pathogens that probably number in the hundreds or thousands. Although on the basis of our data we cannot attribute filovirus infection to individual fish, a possible scenario includes co-infection with FIWIV, OBLV, and KNDV, EGLV, or BPV, or any combination of these viruses. Thamnovirus abundance was low in the sampled European perch, and detection of thamnoviral RNA proved impossible in FFPE tissues. This low abundance, together with the unsuccessful attempt to demonstrate viral RNA in the tissue, suggests that infection in the investigated fish was subclinical rather than the cause of the observed clinical signs.

We also identified a novel hantavirus, Bern perch virus. Hantaviruses are best known as rodent-borne viruses of the mammantavirin genus *Orthohantavirus*, which cause hemorrhagic fever with renal syndrome or hantavirus pulmonary syndrome in humans (*36*) but have also been found in bats and eulipotyphla. Reptile hantaviruses (family *Repantavirinae*, genus *Reptillovirus*) and fish hantaviruses (family *Actantavirinae*, genus *Actinovirus*, and family *Agantavirinae*, genus *Agnathovirus*) (*32*) have only recently been discovered. The fish viruses include the actinoviruses WEMBV, WERSV (*23*), and Wēnlǐng yellow goosefish virus (detected in yellow goosefish [*Lophius litulon*] captured in China [*23*]); the likely actinovirus Aronnax virus, found in pygmy goby (*Eviota zebrina*) purchased at a fish market in Sydney (*31*); and the agnathovirus Wēnlǐng hagfish virus, detected in inshore hagfish (*Eptatretus burgeri*) captured in China (*23*). Similar to our filovirus findings, the discovery of BPV is remarkable because this actinovirus was found in freshwater fish from Europe rather than in marine fish from China or Australia. Actinoviruses have not yet been associated with disease in fish. Using in situ hybridization, we demonstrated, however, high concentrations of BPV RNA in macrophages and endothelial cells in the gills as well as in macrophages in the perivisceral fat tissue of morbid European perch. Other cell types tested negative. Human pathogenic orthohantaviruses predominantly infect macrophages and microvascular endothelial cells of a variety of organs, which leads to increased vascular permeability and severe disease (*11*). It is therefore tempting to speculate that the pathology observed in the gills of the European perch may have resulted in dyspnea, contributing to elevated mortality. In conclusion, our identification of new rhabdoviruses, filoviruses, and hantaviruses in farmed European perch in Switzerland raises concerns about the global distribution, host spectrum, and risks to human and animal health for these viruses.

Appendix Additional methods and results for study of filoviruses and hantaviruses in freshwater fish, Switzerland, 2017.

## References

[1] Food and Agriculture Organization of the United Nations. The state of world fisheries and aquaculture 2018. Meeting the sustainable development goals [cited 2021 Jul 10]. https://www.fao.org/3/i9540en/I9540EN.pdf

[2] Martins CIM, Eding EH, Verdegem MCJ, Heinsbroek LTN, Schneider O, Blancheton JP, et al. New developments in recirculating aquaculture systems in Europe: a perspective on environmental sustainability. Aquacult Eng. 2010;43:83–93. 10.1016/j.aquaeng.2010.09.002

[3] Naylor RL, Hardy RW, Buschmann AH, Bush SR, Cao L, Klinger DH, et al. A 20-year retrospective review of global aquaculture. Nature. 2021;591:551–63. 10.1038/s41586-021-03308-6 33762770

[4] Policar T, Schaefer FJ, Panana E, Meyer S, Teerlinck S, Toner D, et al. Recent progress in European percid fish culture production technology—tackling bottlenecks. Aquacult Int. 2019;27:1151–74. 10.1007/s10499-019-00433-y

[5] Morgan DL, Gill HS, Maddern MG, Beatty SJ. Distribution and impacts of introduced freshwater fishes in Western Australia. N J Mar Freshwater Res. 2004;38:511–23. 10.1080/00288330.2004.9517257

[6] Caruso C, Gustinelli A, Pastorino P, Acutis PL, Prato R, Masoero L, et al. Mortality outbreak by perch rhabdovirus in European perch (*Perca fluviatilis*) farmed in Italy: Clinical presentation and phylogenetic analysis. J Fish Dis. 2019;42:773–6. 10.1111/jfd.12975 30850994

[7] Ruane NM, Rodger HD, McCarthy LJ, Swords D, Dodge M, Kerr RC, et al. Genetic diversity and associated pathology of rhabdovirus infections in farmed and wild perch *Perca fluviatilis* in Ireland. Dis Aquat Organ. 2014;112:121–30. 10.3354/dao02801 25449323

[8] Rupp M, Knüsel R, Sindilariu P-D, Schmidt-Posthaus H. Identification of important pathogens in European perch (*Perca fluviatilis*) culture in recirculating aquaculture systems. Aquacult Int. 2019;27:1045–53. 10.1007/s10499-019-00382-6

[9] Dorson M, Torchy C, Chilmonczyk S, Kinkelin P, Michel C. A rhabdovirus pathogenic for perch, *Perca fluviatilis* L.: isolation and preliminary study. J Fish Dis. 1984;7:241–5. 10.1111/j.1365-2761.1984.tb00929.x

[10] Dannevig BH, Olesen NJ, Jentoft S, Kvellestad A, Taksdal T, Håstein T. The first isolation of a rhabdovirus from perch (*Perca fluviatilis*) in Norway. Bull Eur Assoc Fish Pathol. 2001;21:186–94.

[11] Noack D, Goeijenbier M, Reusken CBEM, Koopmans MPG, Rockx BHG. Orthohantavirus pathogenesis and cell tropism. Front Cell Infect Microbiol. 2020;10:399. 10.3389/fcimb.2020.00399 32903721 PMC7438779

[12] Kauer RV, Koch MC, Hierweger MM, Werder S, Boujon CL, Seuberlich T. Discovery of novel astrovirus genotype species in small ruminants. PeerJ. 2019;7:e7338. 10.7717/peerj.7338 31396439 PMC6679648

[13] Hierweger MM, Werder S, Seuberlich T. Parainfluenza virus 5 infection in neurological disease and encephalitis of cattle. Int J Mol Sci. 2020;21:498. 10.3390/ijms21020498 31941046 PMC7013525

[14] Kuhn JH, Amarasinghe GK, Basler CF, Bavari S, Bukreyev A, Chandran K, et al.; Ictv Report Consortium. ICTV virus taxonomy profile: *Filoviridae.* J Gen Virol. 2019;100:911–2. 10.1099/jgv.0.001252 31021739 PMC7011696

[15] Bào Y, Amarasinghe GK, Basler CF, Bavari S, Bukreyev A, Chandran K, et al. Implementation of objective PASC-derived taxon demarcation criteria for official classification of filoviruses. Viruses. 2017;9:106. 10.3390/v9050106 28492506 PMC5454419

[16] Laenen L, Vergote V, Calisher CH, Klempa B, Klingström J, Kuhn JH, et al. *Hantaviridae*: current classification and future perspectives. Viruses. 2019;11:788. 10.3390/v11090788 31461937 PMC6784073

[17] Walker PJ, Blasdell KR, Calisher CH, Dietzgen RG, Kondo H, Kurath G, et al.; Ictv Report Consortium. ICTV virus taxonomy profile: *Rhabdoviridae.* J Gen Virol. 2018;99:447–8. 10.1099/jgv.0.001020 29465028 PMC12662152

[18] National Center for Biotechnology Information. Pairwise Sequence Comparison (PASC) [cited 2021 Jul 10]. https://www.ncbi.nlm.nih.gov/sutils/pasc/viridty.cgi?textpage=overview

[19] Price MN, Dehal PS, Arkin AP. FastTree 2—approximately maximum-likelihood trees for large alignments. PLoS One. 2010;5:e9490. 10.1371/journal.pone.0009490 20224823 PMC2835736

[20] Kumar S, Stecher G, Li M, Knyaz C, Tamura K. MEGA X: Molecular Evolutionary Genetics Analysis across computing platforms. Mol Biol Evol. 2018;35:1547–9. 10.1093/molbev/msy096 29722887 PMC5967553

[21] Ravasi D, De Respinis S, Wahli T. Multilocus sequence typing reveals clonality in *Saprolegnia parasitica* outbreaks. J Fish Dis. 2018;41:1653–65. 10.1111/jfd.12869 30051543

[22] World Organisation for Animal Health. Aquatic Manual online access [cited 2021 Jul 10]. https://www.oie.int/standard-setting/aquatic-manual/access-online

[23] Shi M, Lin X-D, Chen X, Tian J-H, Chen L-J, Li K, et al. The evolutionary history of vertebrate RNA viruses. Nature. 2018;556:197–202. 10.1038/s41586-018-0012-7 29618816

[24] Hume AJ, Mühlberger E. Distinct genome replication and transcription strategies within the growing filovirus family. J Mol Biol. 2019;431:4290–320. 10.1016/j.jmb.2019.06.029 31260690 PMC6879820

[25] Barr JN, Weber F, Schmaljohn CS. *Bunyavirales*: the viruses and their replication. In: Howley PM, Knipe DM, Whelan SPJ, editors. Fields virology. 7th ed. Philadelphia: Wolters Kluwer/Lippincott Williams & Wilkins; 2020. p. 706–49.

[26] Lauber C, Gorbalenya AE. Partitioning the genetic diversity of a virus family: approach and evaluation through a case study of picornaviruses. J Virol. 2012;86:3890–904. 10.1128/JVI.07173-11 22278230 PMC3302503

[27] International Committee on Taxonomy of Viruses [cited 2021 Jul 10]. https://talk.ictvonline.org/taxonomy

[28] Walker PJ, Siddell SG, Lefkowitz EJ, Mushegian AR, Adriaenssens EM, Alfenas-Zerbini P, et al. Changes to virus taxonomy and to the International Code of Virus Classification and Nomenclature ratified by the International Committee on Taxonomy of Viruses (2021). Arch Virol. 2021;166:2633–48. 10.1007/s00705-021-05156-1 34231026

[29] Kibenge FSB, Godoy MG, editors. Aquaculture virology. Amsterdam: Elsevier; 2016. p. 568.

[30] Geoghegan JL, Di Giallonardo F, Cousins K, Shi M, Williamson JE, Holmes EC. Hidden diversity and evolution of viruses in market fish. Virus Evol. 2018;4:vey031. 10.1093/ve/vey031 30397510 PMC6208713

[31] Geoghegan JL, Di Giallonardo F, Wille M, Ortiz-Baez AS, Costa VA, Ghaly T, et al. Virome composition in marine fish revealed by meta-transcriptomics. Virus Evol. 2021;7:veab005. 10.1093/ve/veab005 PMC7887440 33623709

[32] Kuhn JH, Adkins S, Agwanda BR, Al Kubrusli R, Alkhovsky SV, Amarasinghe GK, et al. 2021 Taxonomic update of phylum Negarnaviricota (Riboviria: Orthornavirae), including the large orders Bunyavirales and Mononegavirales. Arch Virol. 2021;2021:31; Epub ahead of print. 10.1007/s00705-021-05143-6 34463877 PMC8627462

[33] Kurath G, Stone D. Fish rhabdoviruses (*Rhabdoviridae*). In: Bamford D, Zuckerman M, editors. Encyclopedia of Virology. 4th ed. Amsterdam: Elsevier; 2021. p. 324–31.

[34] LaPatra S, Misk E, al-Hussinee L, Lumsden JS. Rhabdoviruses of fish. In: Kibenge FSB, Godoy MG, editors. Aquaculture virology. Amsterdam: Elsevier; 2016. p. 276–97.

[35] Kuhn JH, Amarasinghe GK, Perry DL. *Filoviridae*. In: Howley PM, Knipe DM, Whelan SPJ, editors. Fields virology. 7th ed. Philadelphia: Wolters Kluwer/Lippincott Williams & Wilkins; 2020. p. 449–503.

[36] Kuhn JH, Charrel RN. Arthropod-borne and rodent-borne virus infections. In: Jameson JL, Fauci AS, Kasper DL, Hauser SL, Longo DL, Loscalzo J, editors. Harrison’s principles of internal medicine. 20th ed. Columbus (OH): McGraw-Hill Education; 2018. p. 1489–509.

