# Supplementary material for: Novel Filoviruses, Hantavirus, and Rhabdovirus in Freshwater Fish, Switzerland, 2017
Source: Emerg Infect Dis. 2021 Dec;27(12):3082–91. doi: 10.3201/eid2712.210491 (PMC8632185; doi:10.3201/eid2712.210491)
Supplement: Appendix — Additional methods and results for study of filoviruses and hantaviruses in freshwater fish, Switzerland, 2017. [file 21-0491-Techapp-s1.pdf]

Article DOI: https://doi.org/10.3201/eid2712.210491

Four Filoviruses, 1 Hantavirus, and 1 Rhabdovirus in Freshwater Fish, Switzerland, 2017

Appendix

Methods

Bioinformatics

Reads were quality-trimmed using trimmomatic v. 0.36 (28), and host-derived sequences were removed by aligning reads to the European perch genome (UTU_Pfluv_1.1, Bioproject PRJNA450919) using STAR v. 2.6.0c (*1*). Non-aligned reads were assembled with SPAdes v. 3.12.0 (*2*). The resulting scaffolds were screened for homologies on the nucleotide and amino acid levels using BLASTn v. 2.7.1+ (*3*) against viral nucleotide sequences in GenBank (https://www.ncbi.nlm.nih.gov/genbank/) and DIAMOND v. 0.9.18 (*4*) and against viral protein sequences in UniProt (https://www.uniprot.org/), respectively. (Databases were downloaded on 20 May 2020.)

RT-PCR, RACE, Sanger sequencing

To fill gaps between HTS scaffolds, we reverse-transcribed extracted RNA to cDNA with SuperScript III Reverse Transcriptase (Thermo Fisher Scientific, Waltham, MA, USA) and performed PCR assays with Q5 Hot Start High-Fidelity DNA Polymerase (New England Biolabs, Ipswhich, MA, USA) and scaffold-specific primers (Appendix Table 3) according to the manufacturers' instructions. We gel-purified amplicons using the NucleoSpin Gel & PCR Clean-up Kit (Macherey-Nagel, Oensingen, Switzerland) and sequenced them using a 3730 DNA Analyzer (Thermo Fisher Scientific) with the BigDye Terminator v3.1 Cycle Sequencing Kit (Thermo Fisher Scientific), using standard protocols. We performed 3' and 5' RACE, as described previously on RNA extracted from pooled organs and CNS as well as cell culture supernatants (*5*). We purified RACE products and sequenced them as described above. Resultant data were analyzed with Geneious v 9.1.8 (Biomatters, Auckland, New Zealand).

In Situ hybridization (ISH)

We conducted chromogenic ISH on all of the FFPE tissues used for histopathology. Staining was performed with the RNAscope system (Advanced Cell Diagnostics, Newark, CA, USA). Using the RNAscope 2.5 HD Assay-Brown according to the manufacturer's instructions. ISH probes were designed by the company for EGLV (catalog #590061), BRPV (#590031), FIWIV (#590041), and OBLV (#590051). We counterstained slides with Mayer's hemalum solution (Merck KGaA, Darmstadt, Germany) and mounted them with Aquatex (Merck KGaA). Sections of apparently healthy European perch from a different origin, which we examined for a normal health control, served as negative controls. ISH process controls consisted of brain tissue sections of animals with bovine astrovirus CH13 (BoAstV CH13) infection and a BoAstV CH13-specific RNAscope probe [#406921] tested in parallel to each ISH experiment (*6*).

References

<jrn>1. Dobin A, Davis CA, Schlesinger F, Drenkow J, Zaleski C, Jha S, et al. STAR: ultrafast universal RNA-seq aligner. Bioinformatics. 2013;29:15–21. PubMed https://doi.org/10.1093/bioinformatics/bts635</jrn>

<jrn>2. Bankevich A, Nurk S, Antipov D, Gurevich AA, Dvorkin M, Kulikov AS, et al. SPAdes: a new genome assembly algorithm and its applications to single-cell sequencing. J Comput Biol. 2012;19:455–77. PubMed https://doi.org/10.1089/cmb.2012.0021</jrn>

<jrn>3. Camacho C, Coulouris G, Avagyan V, Ma N, Papadopoulos J, Bealer K, et al. BLAST+: architecture and applications. BMC Bioinformatics. 2009;10:421. PubMed https://doi.org/10.1186/1471-2105-10-421</jrn>

<jrn>4. Buchfink B, Xie C, Huson DH. Fast and sensitive protein alignment using DIAMOND. Nat Methods. 2015;12:59–60. PubMed https://doi.org/10.1038/nmeth.3176</jrn>

<jrn>5. Hierweger MM, Werder S, Seuberlich T. Parainfluenza virus 5 infection in neurological disease and encephalitis of cattle. Int J Mol Sci. 2020;21:498. PubMed https://doi.org/10.3390/ijms21020498</jrn>

<jrn>6. Küchler L, Rüfli I, Koch MC, Hierweger MM, Kauer RV, Boujon CL, et al. Astrovirus-associated polioencephalomyelitis in an alpaca. Viruses. 2020;13:50. PubMed https://doi.org/10.3390/v13010050</jrn>

Appendix Table 1. Results of the bioinformatics pipeline for virus discovery in samples from European perch*

	Scaffold			DIAMOND best protein hit	Amino acid alignment		
Virus family	ID	Length	kmer coverage	Accesssion number|protein|virus	Identity (%)	Length [nt]	Query covera
Hantaviridae	10	6509	3010	A0A2P1GNS4|Large protein|W. red spikefish virus	34.4	2104	94.8
	59	3784	11122	A0A2P1GNS8|Glycoprotein|W. minipizza batfish virus	25.4	836	63.1
	550	2160	15043	A0A2P1GNX7|Nucleoprotein|W. red spikefish virus	30.3	330	42.4
Rhabdoviridae	2058	1454	5.8	Q8UYI1|Nucleocapsid protein|sea trout rhabdovirus	42.3	267	54.8
	4052	1146	3	Q8UY99|Glycoprotein|lake trout rhabdovirus	87.5	80	97.2
	6439	960	5.7	Q8V316|Phosphoprotein|sea trout rhabdovirus	62.2	74	67.5
	8194	873	1.9	K7X7F6|Large protein|perch rhabdovirus	95.2	84	89.4
	10813	771	5.2	Q8V315|Matrix protein|sea trout rhabdovirus	94.8	213	82.9
	10889	768	1.9	A0A0A7|Large protein|eel virus European X	76.4	254	98.8
	12097	731	2.4	K7X7F6|Large protein|perch rhabdovirus	87.2	243	99.7
	37625	394	1	Q8V317|Large protein|lake trout rhabdovirus	93.2	426	87.9
	42117	370	2	K7X7F6|Large protein|perch rhabdovirus	89	354	92.7
	52025	331	1.4	Q8V317|Large protein|lake trout rhabdovirus	93.8	96	99.7
	52641	329	1.1	K7X7F6|Large protein|perch rhabdovirus	84.4	122	98.9
	68156	289	1.3	Q8UY99|Glycoprotein|lake trout rhabdovirus 903/87	77.6	303	94.7
	71803	282	0.96	Q8V313|Large protein|sea trout rhabdovirus	66.7	84	98.8
	89460	256	2.4	K7X7F67|Large sprotein|perch rhabdovirus	98.2	110	99.7
	90246	255	3.8	K7X7F6|Large protein|perch rhabdovirus	82.1	84	98.4
	97442	247	1.7	Q8V313|Polymerase protein |sea trout rhabdovirus	90.8	130	99
Filoviridae	4	14593	23.5	A0A2P1GMM1|Large protein|Huángjiāo virus	50.3	843	17.6
	5	13764	15.2	A0A2P1GMM1|Large protein|Huángjiāo virus	59.8	2145	46.5
	14†	10362	4.8	A0A2P1GMM1|Large protein|Huángjiāo virus	68	1471	42.5
	1005	3259	3	A0A2P1GMM1|Large protein|Huángjiāo virus	47.1	1089	99.9
	10054	3259	3	A0A2P1GMM1|Large protein|Huángjiāo virus	47.1	1089	99.9
	5282†	1945	4.1	A0A2P1GMM1|Large protein|Huángjiāo virus	41	648	99.6
	7085	1729	2.2	A0A2P1GMM5|Nucleoprotein|Huángjiāo virus	38.8	389	66.8
	11776	1389	1.8	A0A2P1GMM1|Large protein|Huángjiāo virus	41.6	334	71.7
	34171†	779	2.6	A0A2P1GMM5|Nucleoprotein|Huángjiāo virus	61.6	250	96.3
	50926	573	3	A0A2P1GMM1|Large protein|Huángjiāo virus	58.4	190	99.5
Other	1	10069	95	A0A1I9QNF6|Capsid protein|marbled eel polyomavirus	27.1	491	13.8
	201	2764	6.8	A0A1S7J028|LargeT|Rousettus aegyptiacus polyomavirus 1	28.6	255	26.6
	44589	359	0.7	A0A2P1GNG4|Polyprotein|Běihǎi rabbitfish_calicivirus	57.4	115	95.3

*Abbreviations: W., Wēnlǐng; put, putative.

†These scaffolds were linked by RT-PCR resulting in the sequence of a novel virus here named Kander virus (KNDV).

Appendix Table 2. Comparison of conserved terminal sequences in genome segments of selected genera of the order *Bunyavirales**

Genus	3′ terminus	5′ terminus
Orthohantavirus	AUCAUCAUCUG...	...AUGAUGAU
Orthobunyavirus	**UCAUCA**CAUGA...	...UCGUG**UGAUGA**
Orthonairovirus	AGAGUUUCU...	...AGAAACUCU
Orthotospovirus	UCUCGUUAG...	...CUAACGAGA
Phlebovirus	UGUCGUUAG...	...CUAACGAGA
Actinovirus	**UCAUCA**UU...	...AA**UGAUGA**
(Species: Perch *actinovirus*, *Bunyavirales*)		

*Adapted from Barr JN, Weber F, Schmaljohn CS. Bunyavirales: the viruses and their replication. In: Howley PM, Knipe DM, Whelan SPJ, editors. Fields virology. 7th ed. Philadelphia, Pennsylvania, USA: Wolters Kluwer/Lippincott Williams & Wilkins; 2020. p. 706-49.

Appendix Table 3. Gene-specific primers for RT-PCRs, Sanger sequencing, and RACE

Name	Sequence	Comment
RT_FiloV_3140F	TGTGAGCTCACCAACCGTAC	Confirmation
RT_FiloV_3483R	GAGCCGTTTCTCCCAAGACA	Confirmation
RT_HantaV_2950F	CCCGGAAGTCCAGAACCTTC	confirmation
RT_HantaV_3269R	CGGTCAGGGAATCATCGGAG	confirmation
RT_RhabdoV_1064F	AAATGCCATTGCCAACACCG	confirmation
RT_RhabdoV_1370R	GTACGCTCCGACAGTGTCTT	confirmation
RT_HantaV_L_493R	AAACTGAAGCTCGATGCCCA	RACE
RT_HantaV_L_322R	CCAGCTGCCCAGGGAATATC	RACE
RT_HantaV_L_5998F	CGTCAGGTCCTGGATCAAGG	RACE
RT_HantaV_L_6190F	CTCCGCTGTGAACATGGTTG	RACE
RT_HantaV_M_541R	TTCTGCCGCCTTTCAAAGC	RACE
RT_HantaV_M_305R	CTCTTGGATCCTGGGTGTCG	RACE
RT_HantaV_M_3273F	ACATAGGCACTGTCCTCAAGC	RACE
RT_HantaV_M_3466F	TTCCGACGAGACCTCCTTCT	RACE
RT_HantaV_S_526R	CAGCCTGTGTTTCCGGAGTA	RACE
RT_HantaV_S_281R	GCTGGATCTGAAGGCAGGAG	RACE
RT_HantaV_S_1656F	CCCAAACAGGTCGGTCATCA	RACE
RT_HantaV_S_1848F	CAAGGTGGTCTCCATGGGG	RACE
RT_RhabdoV_1335F	ATCCAACATGCCCGCAAAGA	connection of contigs
RT_RhabdoV_1684R	TCCCAGCTCCACTAATCCCT	connection of contigs
RT_RhabdoV_2391F	GAATGGAATGGATGCCAGCG	connection of contigs
RT_RhabdoV_2863R	GCCACGACCATCGCATTTTT	connection of contigs
RT_RhabdoV_3358F	GCAATGAGAAAGTGCTGAACCA	connection of contigs
RT_RhabdoV_3750R	CCCATGCCGCAGTTTGATAC	connection of contigs
RT_RhabdoV_3765F	GTAGAGGGGAAGTTGTGCGT	connection of contigs
RT_RhabdoV_4214R	CTGCTGTCGAACGATTGCAC	connection of contigs
RT_RhabdoV_5913F	GTTGGGCGTATGCAGACTCT	connection of contigs
RT_RhabdoV_6225R	GCTCTGCGTTGACAAAGTCA	connection of contigs
RT_RhabdoV_6256F	GATCTTTTCGGCATTGGGGC	connection of contigs
RT_RhabdoV_6559R	TCGAAGATTTTGGTCAGAGGCA	connection of contigs
RT_RhabdoV_6636F	CATGTAGAACGACGGCCCTT	connection of contigs
RT_RhabdoV_7823R	TCCTCGAATTGCCGGATTGT	connection of contigs
RT_RhabdoV_7793F	AGTAGAGTTTCACAATCCGGCA	connection of contigs
RT_RhabdoV_8342R	CAGCGATATCAGGGATTCGGT	connection of contigs
RT_RhabdoV_8780F	GCTGAGAGCCGCTATCACAT	connection of contigs
RT_RhabdoV_9479R	GGACAACGCAGATGCCTTGA	connection of contigs
RT_RhabdoV_10828F	GAGGCTCGACAGGGATCAAG	connection of contigs
RT_RhabdoV_11124R	TCTTCTCCCCTTTGCAACTGA	connection of contigs
RT_Rhabdo_6952F	AGGAGTCAAGGGCAAGGAGA	connection of contigs
RT_Rhabdo_7555R	GCTGACTGGATTGTCTCGTCA	connection of contigs
RT_Rhabdo_6980F	ACGAGCACATCAGTATTGCCA	connection of contigs
RT_Rhabdo_575R	CTCGTTTTGGATCCCCACGA	RACE
RT_Rhabdo_330R	CCAATTCGCTCGGCAATCTG	RACE
RT_Rhabdo_11050F	CGAATCTCTGCTTGCAGTGC	RACE
RT_Rhabdo_11222F	AGGAAGATGGCCACCATTGG	RACE
P_FiloV_13to3360_1435F	CACAGTGGTAAGGGCCATGA	connection of contigs
P_FiloV_13to3360_1884R	CCGGTGTCAACGGCTGTTAT	connection of contigs
P_FiloV_13to5716_12038F	GTCTGCATAGGGAAGGTGGC	connection of contigs
P_FiloV_13to5716_12590R	CTTGTCGCTCCTGAAGACGT	connection of contigs
Perch_FiloV2_296R	TTGCAAGAATGAAGGCACACC	RACE
Perch_FiloV2_511R	AAACCTTGCGGCTTGATTGG	RACE
Perch_FiloV2_14328F	ACGCCTTGGTCAAATCCACA	RACE
Perch_FiloV2_14118F	TTGCTCTTGTGGCAGCAGAA	RACE

Name	Sequence	Comment
Perch_FiloV1_260R	GTCGACTGAGTTGTCTCCCC	RACE
Perch_FiloV1_455R	GTATGGTAATGCCGTTGGTGC	RACE
Perch_FiloV1_13492F	TCCTAGGGAGTCTGCAGAGG	RACE
Perch_FiloV1_13275F	GACAGGTCCGGAGTGAAGTC	RACE

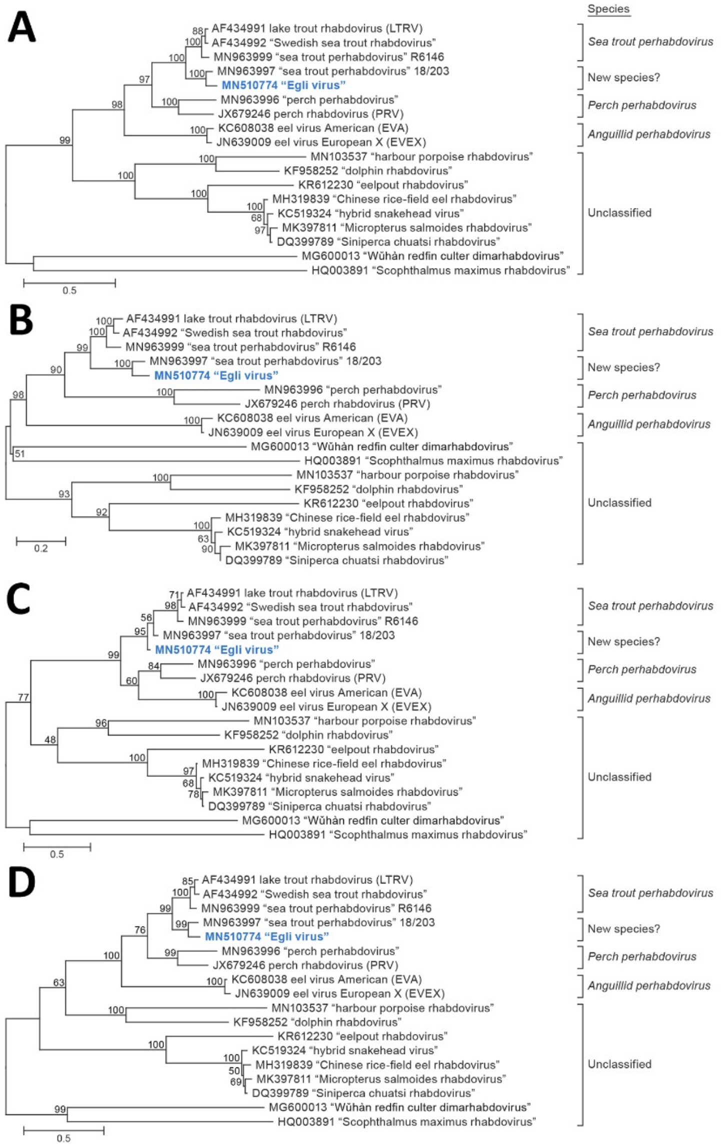

Appendix Figure 1. Maximum-likelihood phylogenetic trees of the nucleotide sequences of Egli virus (EGLV; bold blue) genes with those of viruses belonging to representative members of the genus *Perhabdovirus*. a) nucleoprotein gene (*N*), b) phosphoprotein gene (*P*) c) matrix protein gene (*M*), and d) glycoprotein gene (*G*). Numbers near nodes on the trees indicate bootstrap values. Branches are labeled by GenBank accession number, virus name, and virus name abbreviation in parenthesis. The names of unclassified, likely perhabdoviruses are placed in quotation marks and printed without name abbreviations. The scale (bottom left) indicates the number of substitutions per site, reflected by the branch lengths.

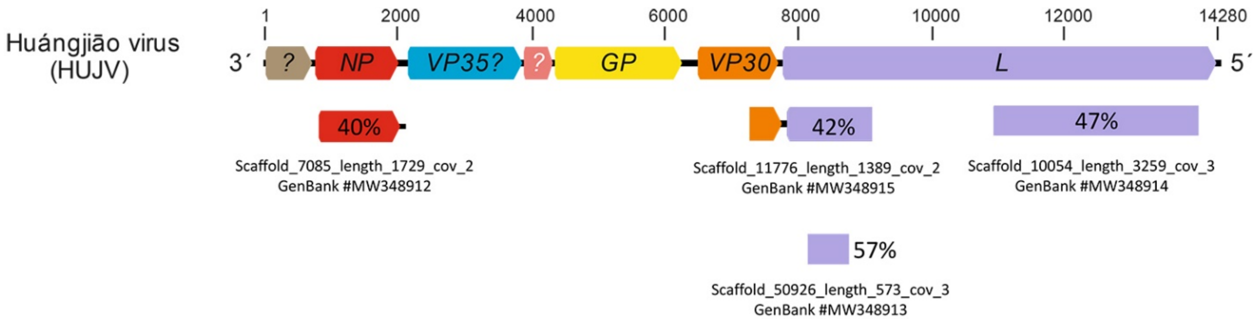

Appendix Figure 2. Mapping of additional scaffolds identified in European perch with hits to Huángjiāo virus (HUJV). The HUJV genome organization is shown schematically on the top and allocated scaffolds (scaffold IDs [shortened] and GenBank accession numbers) to the genomic regions of the encoded proteins with respective hits and their identity at the amino-acid sequence level. Open reading frames (ORFs) are indicated as colored arrows. ORFs encoding HUJV-like proteins (indicated as percentages) are depicted by the same color. *NP*, nucleoprotein gene; *VP35*, polymerase cofactor gene; *GP*, glycoprotein gene; *VP30*, transcriptional activator gene; *L*, large protein gene. Question marks indicate novel ORFs.

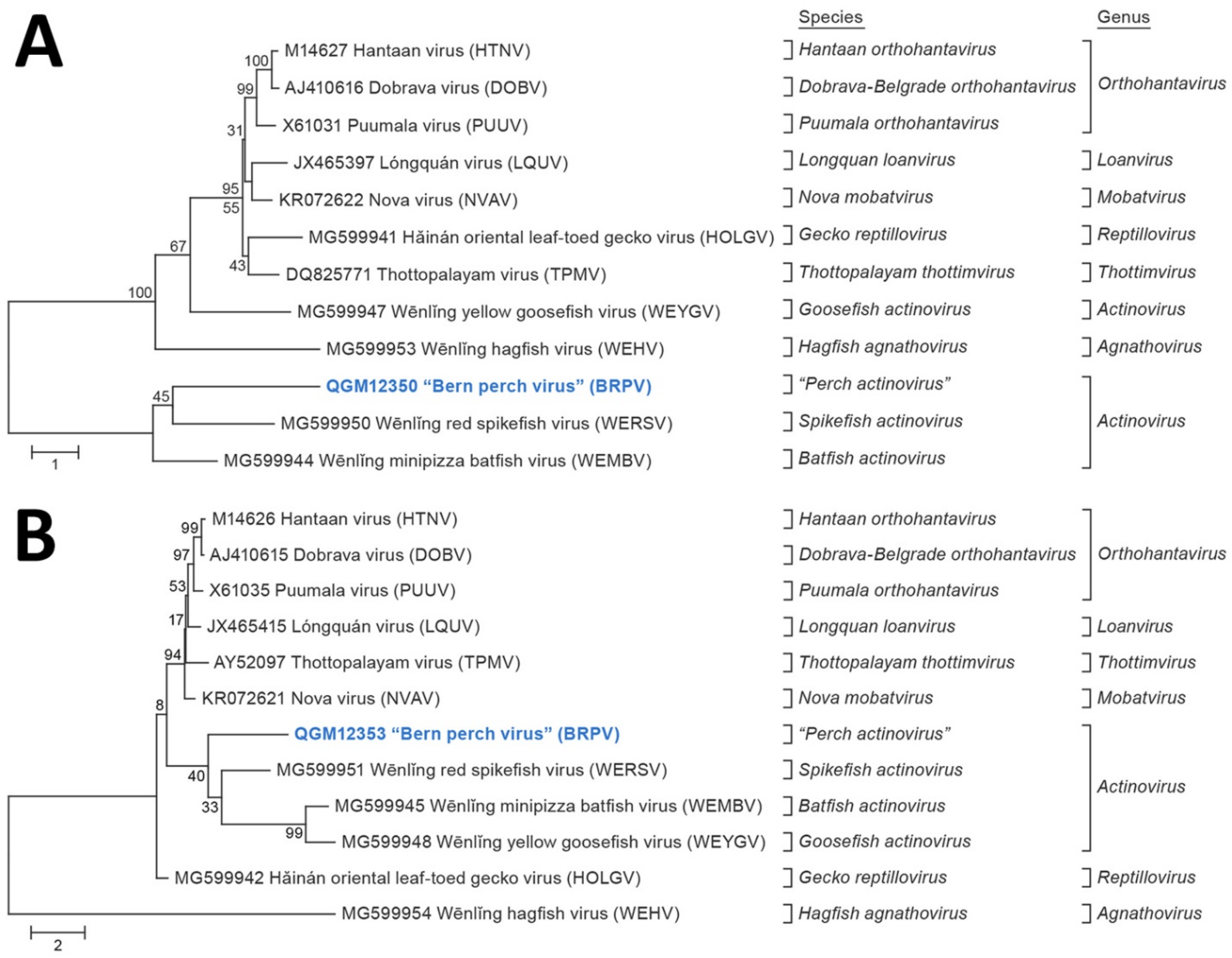

Appendix Figure 3. Maximum-likelihood phylogenetic trees of amino-acid sequences of Bern perch virus (BRPV; bold blue) structural proteins with those of viruses belonging to representative members of the family *Hantaviridae*. a) Glycoprotein precursor (GPC), encoded by the M segment, b) nucleocapsid protein, encoded by the S segment. Numbers near nodes on the trees indicate bootstrap values. Branches are labeled by GenBank accession number, virus name, and virus name abbreviation in parenthesis. Unclassified, likely hantaviruses and officially proposed hantavirus species names are placed in quotation marks. The scale (bottom left) indicates the number of substitutions per site, reflected by the branch lengths.

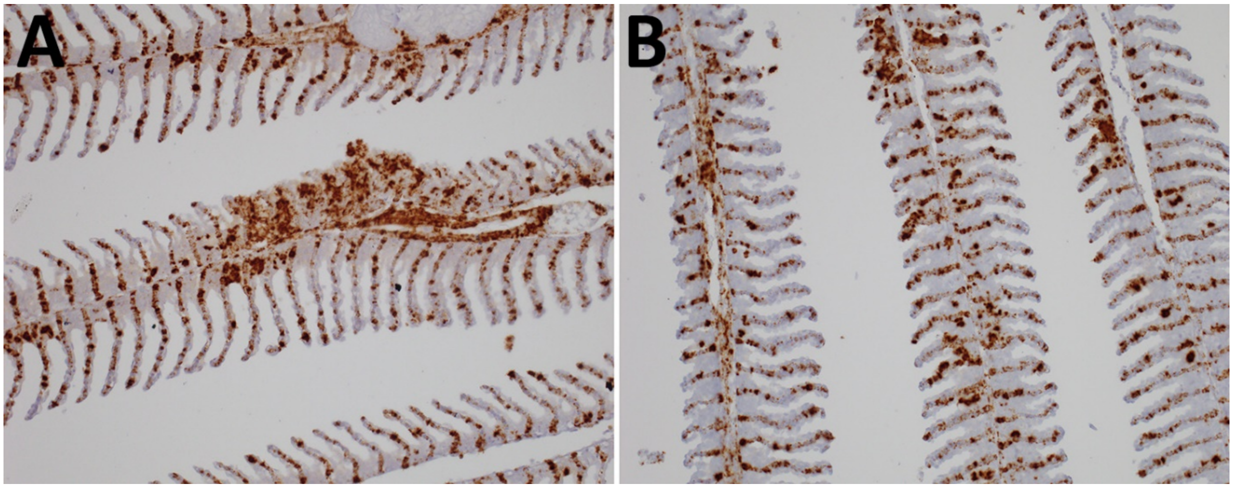

Appendix Figure 4. Detection of Bern perch virus (BRPV) genomic RNA in gills of two individual European perch (A, B) by in situ hybridization (brown labeling).
